# Hospital Annual Meetings

**Published:** 1920-05-22

**Authors:** 


### Hospital Annual Meetings.

### Charing Cross Hospital.

The annual report of the Charing Cross Hospital states that the year 1919 has been signalised by the addition of £28,000 to the invested funds. The allocation to capital of this large amount, of which £20,000 was contributed in one sum by an anonymous donor, was only rendered possible by the continued support of thousands of subscribers. Their generosity has enabled the hospital to pay its way, even in face of the record expenditure of £51,463. It was felt, however, that it would not be possible to raise £50,000 by subscriptions alone, and in future all patients would be asked to contribute to the upkeep of the hospital as far as their means allowed. A tablet commemorating the handsome donation of £20,000, referred to above, has been placed in the main corridor of the hospital. The military wards were closed in February last. During the four and a-half years they were open 3,885 Service men were cared for. The small ward containing twelve beds, to which the Queen gave her name, has, in accordance with Her Majesty's wish, been opened for children. In-patients in 1919 totalled 2,757, as against 3,204 in 1918. New out-patients treated during the period under review were 22,917. In 1918 the number was 19,335. Out-patients attendances in 1919 totalled 90,126, as compared with 81,212 a year previously. The average number of beds occupied daily last year was 183, as compared with 250 in 1918. The reduction was attributable to the necessity of closing the military wards for renovation and repair after they ceased to be occupied.

### Royal National Hospital for Consumption.

At the Annual Meeting of Governors of the Royal National Hospital for Consumption and Diseases of the Chest, Ventnor, held recently, it was reported that during 1919, which completes fifty years' record of work since the institution was opened for the reception of patients, 879 persons were under treatment, as compared with 803 a year previously. The average length of stay of each case treated to a conclusion was eighty-five days. The total number of patients received since the inception of the hospital was 30,830. Although the annual subscriptions of £3,773 during the past year amounted to a higher figure than ever before, the excess of expenditure over income was £2,224. The ordinary expenditure compared with 1913 increased by 57 per cent. It is a matter of keen regret to the Board that the hospital has never yet succeeded in obtaining a grant from the King's Fund in recognition of the benefits conferred on London patients, who form 50 per cent. of the whole of the cases received at Ventnor. An addition has been made to the property at Ventnor through the acquisition on favourable terms of rather more than six acres of land. The charge to a patient has been increased from 15s. to 20s. a week, and that to a public authority for patients sent to the hospital from 42s. to 49s. a week. Beds to the average number of 167 were occupied daily in 1919, as against 158 a year previously.

